# Findings From the Gen Silent Survey Project: A Mixed Methods Pre-Post Design

**DOI:** 10.1093/geroni/igaf122.1787

**Published:** 2025-12-31

**Authors:** Alison Rataj, Kristen Porter, Beth Dugan

**Affiliations:** College of Health and Human Services, Center on Aging and Community Living, Durham, New Hampshire, United States; University of Massachusetts Boston, Boston, Massachusetts, United States; University of Massachusetts Boston, Boston, Massachusetts, United States

## Abstract

Providers lack access to accessible, virtual, cost-effective training resources to safely serve LGBTQ+ older adults. The purpose of this study was to evaluate change in knowledge, attitudes, and anticipated behaviors of providers after watching the film Gen Silent through a quasi-experimental pretest-posttest survey design. Primary data was collected from aging service providers (e.g., long-term care ombudsmen, nurses, social workers, site supervisors) across one in-person (pre-pandemic) and two-virtual (during pandemic) screenings. Data were analyzed using the Wilcoxon signed-rank test as well as, linear and logistic regression models. Most participants (79%) improved their survey score after watching Gen Silent. Scores on eight out of nine measures of knowledge, attitudes, and anticipated behavior improved after watching the film. Between group differences were found. Baby Boomers were significantly more likely to show improvement on posttest scores (B = 0.19, p < .05) compared to participants from other generations. Additionally, LGBTQ+ individuals were significantly more likely to score higher on the pretest (B = 0.26 p < .01), similarly, participants caring for a LGBTQ+ individual were more likely to have higher pretest scores (B = 0.20, p >.05). Eighty-three (83%) of participants indicated that their thoughts and views changed after watching the film due to deepened understanding of LGBTQ+ aging issues. Findings imply that the use of Gen Silent is a relevant educational tool that can be utilized virtually to increase providers’ awareness of the lived experiences of, and empathy toward, LGBTQ+ older adults.

